# P2X and P2Y receptor signaling in red blood cells

**DOI:** 10.3389/fmolb.2015.00060

**Published:** 2015-10-28

**Authors:** Ronald Sluyter

**Affiliations:** ^1^School of Biological Sciences, University of WollongongWollongong, NSW, Australia; ^2^Centre for Medical and Molecular Bioscience, University of WollongongWollongong, NSW, Australia; ^3^Illawarra Health and Medical Research InstituteWollongong, NSW, Australia

**Keywords:** erythrocyte, red blood cell, adenosine triphosphate, purinergic receptor, P2X1 receptor, P2X7 receptor, P2Y1 receptor, P2Y13 receptor

## Abstract

Purinergic signaling involves the activation of cell surface P1 and P2 receptors by extracellular nucleosides and nucleotides such as adenosine and adenosine triphosphate (ATP), respectively. P2 receptors comprise P2X and P2Y receptors, and have well-established roles in leukocyte and platelet biology. Emerging evidence indicates important roles for these receptors in red blood cells. P2 receptor activation stimulates a number of signaling pathways in progenitor red blood cells resulting in microparticle release, reactive oxygen species formation, and apoptosis. Likewise, activation of P2 receptors in mature red blood cells stimulates signaling pathways mediating volume regulation, eicosanoid release, phosphatidylserine exposure, hemolysis, impaired ATP release, and susceptibility or resistance to infection. This review summarizes the distribution of P2 receptors in red blood cells, and outlines the functions of P2 receptor signaling in these cells and its implications in red blood cell biology.

## Introduction

It is well-established that extracellular adenosine triphosphate (ATP) and other nucleotides function through cell surface purinergic receptors to mediate numerous signaling events in all cell types (Burnstock and Knight, 2004). Purinergic receptors that respond to extracellular nucleotides are termed P2 receptors, and comprise P2X and P2Y receptor subtypes (Burnstock and Kennedy, 1985). P2X receptors are trimeric ATP-gated cation channels that mediate the rapid flux of Na^+^, K^+^, and Ca^2+^, with some members also mediating the rapid flux of organic ions (Kaczmarek-Hajek et al., 2012). In mammals, seven P2X receptor subunits exist (P2X1–P2X7), which combine to form either homomeric or heteromeric receptors (Kaczmarek-Hajek et al., 2012). P2Y receptors are G protein-coupled receptors and modulate various signaling events including adenylyl cyclase, phospholipase C, and ion channel activation (Abbracchio et al., 2006). To date eight P2Y receptors have been identified in mammals (P2Y1, P2Y2, P2Y4, P2Y6, and P2Y11–P2Y14). Unlike P2X receptors, some P2Y receptor subtypes are preferentially activated by nucleotides other than ATP, such as P2Y2 and P2Y13, which are preferentially activated by uridine triphosphate (UTP) and adenosine diphosphate (ADP), respectively. Furthermore, ADP is an agonist of many P2Y receptor subtypes (Abbracchio et al., 2006).

P2 receptors are present on all blood cells (Burnstock, 2015). In particular, P2X7 has well-established roles on leukocytes (Bartlett et al., 2014), while P2Y1 and P2Y12 have well-defined functions on platelets (Gachet, 2008). P2 receptors also play important roles in hematopoietic stem cells (Rossi et al., 2012). Collectively, P2 receptor activation contributes to inflammation (Idzko et al., 2014a), and vascular and blood disease (Idzko et al., 2014b), as evidenced by studies of P2 receptor-deficient mice (Labasi et al., 2002; Stachon et al., 2014). Moreover, it is becoming apparent that P2 receptors have important roles in red blood cells (RBCs), a salient point given the importance of ATP release from RBCs within the vasculature (Sprague and Ellsworth, 2012). This review aims to provide an overview of the distribution of P2 receptors on RBC progenitors and mature RBCs (erythrocytes), and to outline the functions of P2 receptor signaling in these cell types. Other aspects relevant to purinergic signaling in RBCs including ATP release, ectonucleotidases and P1 receptors have been subject to earlier reviews (Huber, 2012; Burnstock, 2015).

## Distribution of P2 receptors in progenitor red blood cells

P2 receptors have been identified in progenitor RBCs from humans and mice. RT-PCR of human erythroid progenitors, derived by culture of CD34^+^ cells, reveals mRNA for P2X1, P2X4, P2X7, and P2Y1, but not P2Y2, P2Y4, and P2Y6 (Hoffman et al., 2004). Further, quantitative PCR reveals high amounts of P2Y13 mRNA, and lower amounts of P2Y1 and P2Y12 in human reticulocytes (Wang et al., 2005). RT-PCR, and immunoblotting and immunolabeling reveal P2X7 mRNA and protein, respectively in murine erythroleukemic (MEL) cells (Constantinescu et al., 2010), a model of progenitor RBCs (Friend et al., 1971). Finally, RT-PCR demonstrates P2Y1, P2Y2, and P2Y12, but not P2Y4, mRNA in murine bone marrow erythroblasts (Paredes-Gamero et al., 2006).

## P2X receptor function in progenitor red blood cells

Evidence for functional P2X receptors in progenitor RBCs is limited to P2X7, and then only in MEL cells. Over 30 years ago, ATP was shown to induce Na^+^, K^+^, and Ca^2+^ fluxes, and death in MEL cells (Chahwala and Cantley, 1984), although the role of purinergic receptors in these processes was not considered at the time. Subsequently, it was demonstrated that P2X7 activation mediates ATP-induced rapid dye uptake and apoptosis in MEL cells (Constantinescu et al., 2010). A role for P2X7 in ATP-induced Na^+^, K^+^, and Ca^2+^ fluxes was not examined, but this study indicates that the initial ATP-induced cation fluxes observed in the earlier study (Chahwala and Cantley, 1984) were most probably mediated by P2X7. P2X7 activation in MEL cells induces rapid phosphatidylserine (PS) exposure, microparticle release, apoptosis (Constantinescu et al., 2010) and reactive oxygen species formation (Wang and Sluyter, 2013). In contrast to other cell types (Bartlett et al., 2013), reactive oxygen species formation is not essential for P2X7-induced apoptosis in MEL cells, but requires p38 mitogen-activated protein kinase and caspase activation (Wang and Sluyter, 2013). The role of P2X7 activation in progenitor RBCs remains to be determined, but may cause the removal of damaged RBC progenitors to prevent development of anemia, leukemia, or autoimmunity.

## P2Y receptor function in progenitor red blood cells

The presence of functional P2Y receptors in progenitor RBCs is mainly limited to P2Y1. ATP, ADP, and UTP induce release of Ca^2+^ from intracellular stores within murine bone marrow erythroblasts suggesting the presence of functional P2Y receptors in these cells (Paredes-Gamero et al., 2006). Although the identity of the receptors responsible for the ATP- and UTP-induced responses were not resolved, the ADP-induced release of intracellular Ca^2+^ was caused by P2Y1 activation (Paredes-Gamero et al., 2006). Functional P2Y1 may also be present in in human progenitor RBCs, but direct evidence is sparse. ADP, an agonist of P2Y1 but also other P2Y receptors (Abbracchio et al., 2006), can cause the release of intracellular Ca^2+^ within human erythroid progenitors generated from peripheral blood (Porzig et al., 1995). The physiological roles of P2Y1 activation in progenitor RBCs remain to be explored.

## Distribution of P2 receptors in red blood cells

P2 receptors have been identified in RBCs from various species. Quantitative PCR reveals high amounts of P2Y13 mRNA, low amounts of P2X1, P2X4, P2X7, and P2Y2, and even lower amounts of P2Y1, P2Y4, P2Y6, P2Y11, and P2Y12 in human RBCs (Wang et al., 2005). Immunoblotting demonstrates the presence of P2X1 and P2X7 protein in human, canine and murine RBCs (Sluyter et al., 2007a; Skals et al., 2009), and P2Y1 protein in human RBCs (Tanneur et al., 2006). Immunolabeling confirms the presence of P2X7 protein in RBCs from humans (Sluyter et al., 2004) and dogs (Sluyter et al., 2007a), as well as P2Y1 (Tanneur et al., 2006), and to a lesser extent P2X2 (Sluyter et al., 2004) and P2Y2 (Tanneur et al., 2004) in human RBCs.

## P2X receptor function in red blood cells

P2X1 and P2X7 mediate bacterial toxin-induced lysis of RBCs from various species. Both P2X1 and P2X7, but not P2Y1 or P2Y2, mediate *Escherichia coli* α-hemolysin-induced lysis of human, murine and equine RBCs (Skals et al., 2009). This effect is primarily mediated by P2X1 in murine RBCs, but P2X7 in human RBCs (Skals et al., 2009) suggesting that these receptors are differentially expressed in RBCs from these two species. In contrast, python RBCs are resistant to α-hemolysin (Larsen et al., 2011). α-Hemolysin also induces cell shrinkage of and PS exposure on human RBCs, and the subsequent phagocytosis of these cells by human THP-1 monocytes (Fagerberg et al., 2013) implying that RBCs exposed to this toxin can be cleared from the circulation. Through the use of the P2X1 antagonist MRS2159 and P2X7 antagonists, this study also indicated that both P2X1 and P2X7 mediate α-hemolysin-induced PS exposure (Fagerberg et al., 2013). However, MRS2159 is also a potent antagonist of human P2X7 (Sophocleous et al., 2015) leaving open the possibility that P2X7, but not P2X1, mediates this event in human RBCs.

P2X1 and P2X7 also mediate *Staphylococcus aureus* α-toxin-induced lysis of murine and equine RBCs (Skals et al., 2011) and *Aggregatibacter actinomycetemcomitans* leukotoxin A-induced lysis of human RBCs (Munksgaard et al., 2012). The latter study also demonstrated that leukotoxin A induced shrinkage of and PS exposure on RBCs (Munksgaard et al., 2012), although the role of P2X1 or P2X7 in these processes was not elucidated. P2X7 activation also mediates *Actinobacillus pleuropneumoniae* ApxIA toxin-induced lysis of ovine RBCs (Masin et al., 2013). In contrast, P2X receptor activation was not required for *Bordetlla pertussis* adenylate cyclase toxin-induced lysis of ovine RBCs (Masin et al., 2013). Collectively, the authors concluded that involvement of P2X receptor activation in hemolysis could be regulated by toxin pore size, with ApxIA hemolysin forming larger pores (~2.4 nm) than adenylate cyclase toxin (~0.7 nm) (Masin et al., 2013). Finally, *E. coli* shiga toxin can induce microvesicle release from human RBCs; a process blocked by broad-spectrum P2 receptor antagonists (Arvidsson et al., 2015), however the specific P2 receptors involved remain unknown.

Complement can also induce lysis of human, murine, and ovine RBCs via P2X1 and P2X7 activation (Hejl et al., 2013). Notably, the P2 receptor antagonist suramin was originally shown to impair complement-mediated lysis of human and guinea pig RBCs, although this effect appeared to be due to suramin directly binding complement components (Fong and Good, 1972). Nevertheless, a role for P2X receptor activation in this early study cannot be excluded.

**Figure 1:**
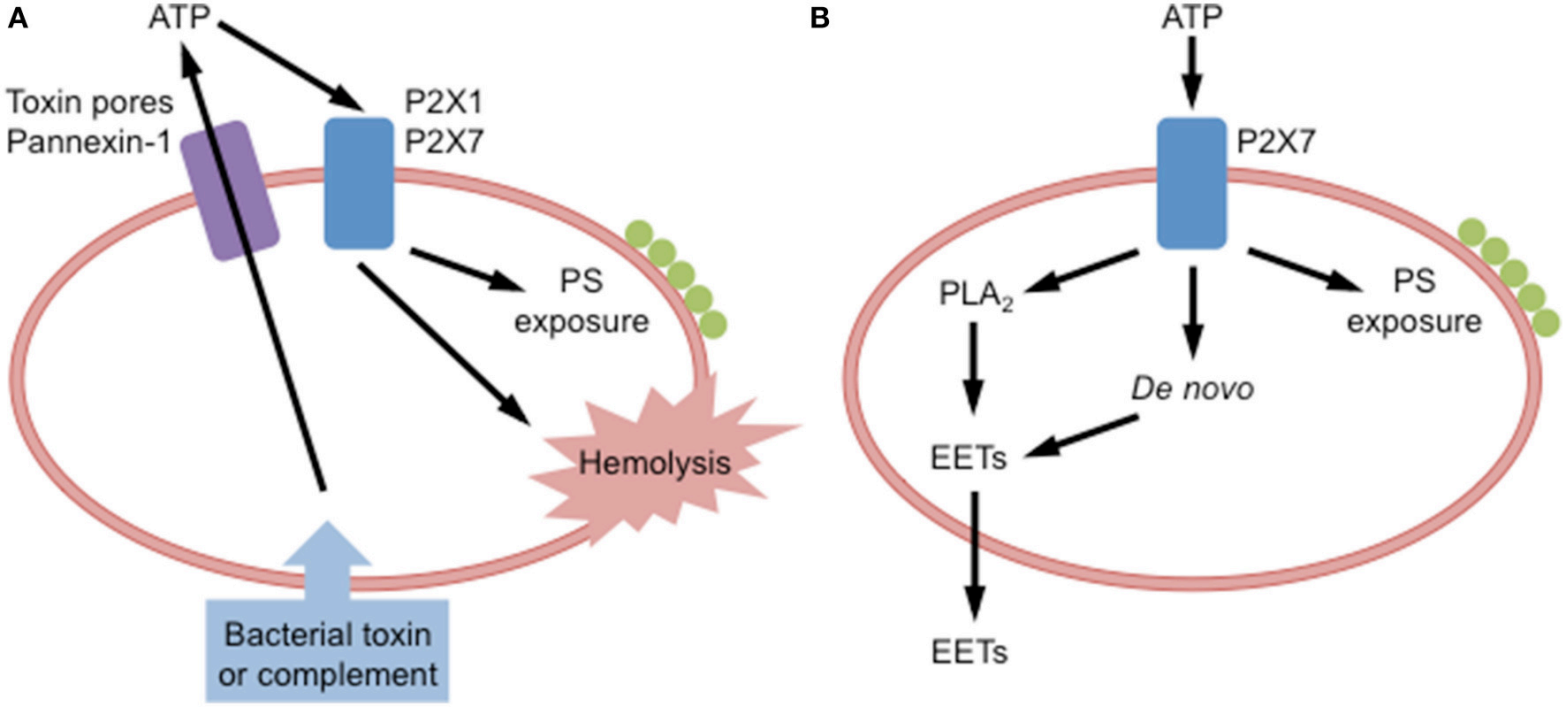
**P2X receptor activation in red blood cells**. **(A)** Binding of bacterial toxins or complement to red blood cells causes adenosine triphosphate (ATP) release via toxin pores and pannexin-1. Released ATP can activate P2X1 and P2X7 receptors on these cells to induce phosphatidylserine (PS) exposure and hemolysis. **(B)** Extracellular ATP can activate P2X7 receptors on red blood cells to induce PS exposure, or formation of epoxyeicosatrienoic acids (EETs), via phospholipase A_2_ (PLA_2_) acting on stored phospholipids or via *de novo* synthesis, and subsequent EETs release.

Bacterial toxin-induced and complement-induced hemolysis involves the release of ATP acting on P2X receptors in an autocrine or paracrine fashion (Figure 1A). ATP scavenging enzymes impair α-hemolysin, α-toxin and leukotoxin A-induced lysis of RBCs (Skals et al., 2009; Munksgaard et al., 2012), and complement-induced hemolysis (Hejl et al., 2013) supporting the concept that released ATP activates P2X receptors. Originally it was thought that hemichannel pannexin-1, which can mediate ATP release from RBCs (Locovei et al., 2006), was responsible for the above ATP release (Skals et al., 2011), as pannexin-1 antagonists prevented toxin-induced hemolysis (Skals et al., 2009, 2011; Munksgaard et al., 2012). However, recent findings indicate that α-hemolysin and leukotoxin A induce ATP release from RBCs by forming toxin pores rather than via pannexin-1 (Skals et al., 2014). Thus, current evidence suggests that bacterial toxins directly from pores in RBCs to allow ATP release, which then acts on P2X1 and P2X7 to mediate hemolysis. The mechanism by which complement causes ATP release remains to be resolved. However, recent data indicates that ligation of complement receptor 1 on human RBCs mediates ATP release (Melhorn et al., 2013). Both bacterial toxin-induced and complement-induced hemolysis pose potential health problems during certain bacterial infections and in diseases associated with prolonged complement activation.

Direct evidence for functional P2X7 in RBCs was first demonstrated for human RBCs. P2X7 activation mediates Na^+^ and Rb^+^ (K^+^) fluxes, as well as choline^+^ uptake in human RBCs (Sluyter et al., 2004; Stevenson et al., 2009). Moreover, P2X7 activation can induce Rb^+^ efflux and choline^+^ uptake in canine RBCs (Sluyter et al., 2007a; Shemon et al., 2008; Stevenson et al., 2009). The ability of ATP to induce cation fluxes in canine RBCs was first observed in 1972 (Parker and Snow, 1972) and subsequently by others that same decade (Elford, 1975; Romualdez et al., 1976). These early investigations did not attribute this effect to purinergic signaling, despite the establishment of this concept by Burnstock also in 1972 (Burnstock, 1972). Nevertheless, it is evident from the initial observations (Parker and Snow, 1972) that ATP induced Na^+^ or K^+^ fluxes in canine RBCs in a manner characteristic of P2X7 activation, and with a time course and order of magnitude near identical to that of ATP-induced Rb^+^ fluxes in canine RBCs observed some 30 years later (Sluyter et al., 2007a). Thus, this original observation that ATP mediates cation fluxes in canine RBCs (Parker and Snow, 1972) remains one of the earliest known reports of functional P2X7 in any cell type. Notably, relative P2X7 activity in canine RBCs is up to 100-fold greater than that observed in human RBCs (Sluyter et al., 2007a; Stevenson et al., 2009). This increased P2X7 activity in canine RBCs corresponds to increased amounts of P2X7 in canine RBCs compared to human RBCs (Sluyter et al., 2007a). The physiological significance of this observation remains unknown, as does the relative amount or activity of P2X7 on RBCs between other species.

P2X7 activation induces PS exposure in human RBCs (Figure 1B) either freshly isolated from peripheral blood (Sluyter et al., 2007b) or following cold storage for up to 6 weeks (Sophocleous et al., 2015). Notably, the amount of P2X7-induced PS exposure varies between donors (Sophocleous et al., 2015). This is mostly likely due to single nucleotide polymorphisms in the *P2RX7* gene that code for loss or gain of P2X7 function (Sluyter and Stokes, 2011). Consistent with this concept, ATP-induced cation fluxes and PS exposure are reduced in RBCs from subjects coding loss-of-function *P2RX7* gene mutations (Sluyter et al., 2004, 2007b), while gain-of-function mutations correspond with augmented ATP-induced cation fluxes in RBCs (Stokes et al., 2010). ATP can also induce PS exposure and hemolysis in canine RBCs (Sluyter et al., 2007a), but direct evidence for P2X7 in these processes is lacking. Furthermore, mutations that alter receptor function are found in the *P2RX7* gene of dogs (Spildrejorde et al., 2014), but it remains to be determined if these mutations alter P2X7-mediated events in canine RBCs. The physiological significance of P2X7-mediated PS exposure in RBCs remains unknown, but the propensity of human RBCs to undergo PS externalization does not change with *in vitro* or *in vivo* aging (Sophocleous et al., 2015). This suggests that P2X7-mediated PS exposure in RBCs does not play a role in the normal removal of senescent RBCs, but perhaps in the removal of RBCs following cell stress or damage, or in diseased states.

P2X7 activation induces epoxyeicosatrienoic acid (EET) release from rat RBCs (Jiang et al., 2007). This release of EETs is partly dependent on phospholipase A_2_ stimulation, but not hemolysis (Jiang et al., 2007). In combination with earlier data (Jiang et al., 2005), EETs released downstream of P2X7 activation represent both EETs generated from stored phospholipids and from *de novo* synthesis (Jiang et al., 2007) (Figure 1B). EETs are eicosanoids that mediate a variety of functions within the circulation including vasodilation (Jiang et al., 2010), thus P2X7-mediated EET release may amplify the circulatory responses mediated by extracellular ATP (Jiang et al., 2007, 2010). It remains to be determined if P2X7 activation can induce EET release from RBCs of other species.

Functional P2X receptors have been reported in non-mammalian RBCs. During hypotonic swelling, ATP is released from *Necturus* salamander RBCs to stimulate regulatory volume decrease in these cells (Light et al., 1999). Pharmacological approaches indicated that this receptor is most likely a P2X2 homolog (Light et al., 2001), while other studies showed that activation of this P2X2-like receptor mediates Ca^2+^ influx during hypotonic swelling of *Necturus* RBCs (Light et al., 2003). ATP release also regulates volume decreases during hypotonic swelling of skate RBCs (Goldstein et al., 2003), but direct evidence for P2 receptors in this process is lacking. In contrast, a P2X-like receptor stimulates regulatory volume decrease in alligator cells (Wormser et al., 2011). Activation of this receptor stimulates Ca^2+^ influx to activate phospholipase A_2_ and arachidonic acid release to increase K^+^ permeability and volume recovery (Wormser et al., 2011). At present there is no evidence that P2X receptor activation stimulates regulatory volume decrease in mammalian RBCs. Finally, functional P2X receptors have been identified in RBCs from other reptiles. Activation of P2X-like receptors in RBCs from Iguania lizards causes an influx of Ca^2+^ (Bagnaresi et al., 2007; Beraldo and Garcia, 2007). In contrast, RBCs from Scleroglossa lizards do not appear to express functional P2X receptors, but rather a P2Y4-like receptor that causes intracellular Ca^2+^ release following activation (Sartorello and Garcia, 2005).

## P2Y receptor function in red blood cells

The first direct evidence for functional P2 receptors in RBCs was established through a series of studies demonstrating the presence of P2Y1 in turkey RBCs (see Boyer et al., 1996). A P2Y receptor was initially identified in membranes of turkey RBCs (Harden et al., 1988) and then in whole turkey RBCs (Berrie et al., 1989; Boyer et al., 1989). Combined, these studies showed that activation of this receptor stimulates phosphatidylinositol 4,5-biphophate hydrolysis and phospholipase C activation (Harden et al., 1988; Berrie et al., 1989; Boyer et al., 1989). Subsequent cloning identified this receptor as the turkey homolog of human and chick P2Y1 (Filtz et al., 1994).

**Figure 2:**
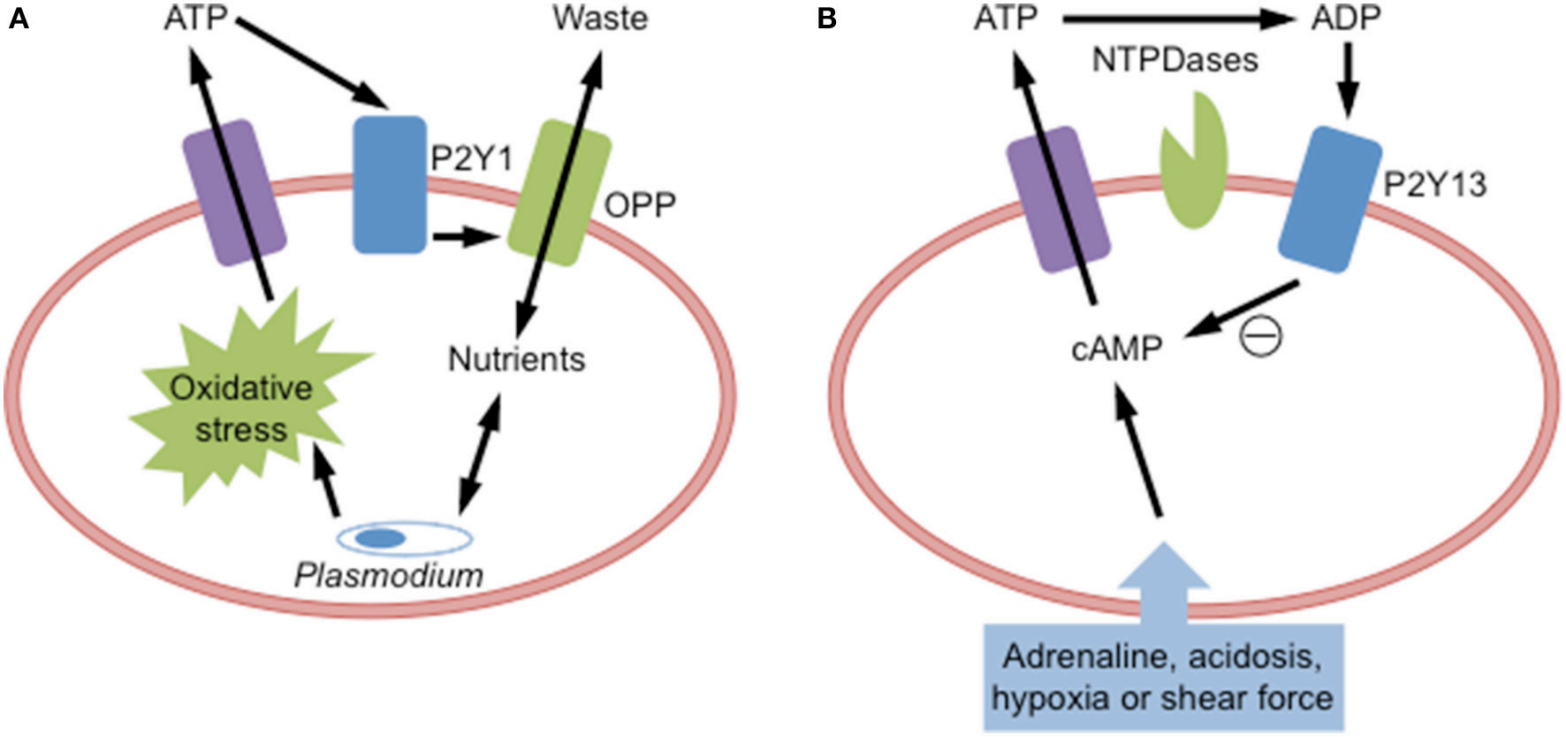
**P2Y receptor activation in red blood cells**. **(A)** *Plasmodium* malarial parasite infection of red blood cells causes oxidative stress to induce adenosine triphosphate (ATP) release, which activates P2Y1 receptors to open an osmolyte permeability pathway (OPP) to facilitate parasite growth by supplying incoming nutrients and removing outgoing metabolic waste products. **(B)** Cellular stress (acidosis, adrenaline, hypoxia, or shear force) of red blood cells increases intracellular cyclic adenosine monophosphate (cAMP) to cause ATP release. Released ATP can be degraded by ectonucleotidases (NTPDases) to adenosine diphosphate (ADP), which then activates P2Y13 receptors to reduce cAMP and prevent further ATP release.

Functional P2Y1 is also present in human and murine RBCs, where it plays a role in promoting malaria parasite development. *Plasmodium* infection of human or murine RBCs results in ATP release (Tanneur et al., 2006; Akkaya et al., 2009) and the subsequent activation of P2Y1 to open an osmolyte permeability pathway (Tanneur et al., 2006), which potentially promotes parasite development through the supply of nutrients and removal of metabolic waste products (Kirk, 2001). Similar findings where also observed with oxidized RBCs suggesting that parasite-derived oxidative stress is involved in the induction of this P2Y1-induced osmolyte permeability pathway (Tanneur et al., 2006) (Figure 2A). Studies of P2 receptor activation in malaria-infected RBCs however are complicated by evidence that *Plasmodium* malaria parasites also express functional P2 receptors (Levano-Garcia et al., 2010; da Cruz et al., 2012).

RBCs may express functional P2Y12, but direct evidence is limited. In human RBCs, the P2Y12 antagonist ticagrelor inhibits adenosine uptake (van Giezen et al., 2012) and induces the release of ATP, which can be subsequently degraded to adenosine (Ohman et al., 2012). Further, ticagrelor augments cardiac blood flow in dogs (van Giezen et al., 2012) indirectly suggesting that P2Y12 may be present on canine RBCs. Combined these studies suggested that ticagrelor may provide cardiovascular benefits in addition to ADP-induced platelet aggregation.

Functional P2Y13 is present on RBCs, where it negatively regulates ATP release from these cells (Wang et al., 2005). Activation of this receptor by ADP impairs the release of ATP from human RBCs (Figure 2B). Moreover, intracoronary injection of the P2Y13 agonist 2-methylthio-ADP into pigs reduces the amount of circulating ATP (Wang et al., 2005). Further evidence defining a role for this receptor in this feedback mechanism is wanting.

## Conclusions

Various P2 receptors are present in progenitor and mature RBCs. Evidence for functional P2 receptors in primary progenitor RBCs remains to be fully explored, but studies of MEL cells indicate that P2X7 can mediate microparticle release, reactive oxygen species formation, and apoptosis. A larger body of evidence is available for the presence of functional P2 receptors in mature RBCs, with P2X1, P2X7, P2Y1, and P2Y13 being the major P2 receptor subtypes present. In RBCs, P2X1 and P2X7 mediate ATP-induced PS exposure, hemolysis, and eicosanoid release. P2Y1 facilitates malaria parasite development within RBCs, while P2Y13 functions to negatively regulate ATP release from RBCs. Despite these findings, further investigations are required to fully define the role of P2 receptors in RBCs.

## Author contributions

RS conceived and wrote the manuscript, and prepared the figures.

### Conflict of interest statement

The author declares that the research was conducted in the absence of any commercial or financial relationships that could be construed as a potential conflict of interest.
